# Evidence-Based Veterinary Medicine: A Tool for Evaluating the Healing Process After Surgical Treatment for Cranial Cruciate Ligament Rupture in Dogs

**DOI:** 10.3389/fvets.2019.00065

**Published:** 2019-03-05

**Authors:** Stefania Pinna, Carlotta Lambertini, Lisa Grassato, Noemi Romagnoli

**Affiliations:** Department of Veterinary Medical Sciences, Alma Mater Studiorum, University of Bologna, Bologna, Italy

**Keywords:** questionnaire, index, healing, stifle, cranial cruciate ligament, dog

## Abstract

This study aims to validate a tool, the Bologna healing stifle injury index (BHSII), for the evaluation of the clinical picture and the healing after surgical treatment for cranial cruciate ligament (CCL) rupture. The study included 158 client-owned dogs with CCL rupture and 20 healthy dogs. The BHSII is a questionnaire made up of 34 multiple-choice questions, divided into a part directed to the clinician and a part for the dog's owners. It was applied twice in the healthy dogs in order to test and retest the device. It was evaluated for reliability, validity, and responsiveness to clinical changes involving the dogs treated at the time of surgery, and 1, 3, and 6 months postoperatively. Statistical analyses were performed and the intraclass correlation coefficient test was ≥0.9 and the Cronbach-α was 0.84 suggesting good stability and good internal consistency of the tool. The area under the curve of the receiver operating characteristic curve was >0.9, indicative of the high accuracy of this tool. The clinician survey correlated with the owner questionnaire. In dogs with CCL rupture, the scores of the BHSII increased significantly postoperatively as compared with baseline. In conclusion, this clinical study proved the reliability, validity, and responsiveness of the BHSII. The results achieved from the BHSII provided an instantaneous, collective complete vision of the healing process of the stifle joints treated. It can be considered a valid tool for collecting data and for assessing successful surgical treatment in clinical practice.

## Introduction

Cranial cruciate ligament (CCL) rupture is one of the most common canine orthopedic injuries, and a very frequent cause of pain and lameness in dogs (1–4).

The treatment of CCL rupture aims to anatomically or mechanically resolve joint instability and to provide long-term functioning of the affected hindlimb. Over time, many surgical techniques, classified as intracapsular, extracapsular, and osteotomies, have been reported, studied and compared in order to identify the gold standard (5, 6). However, according to Aragon and Budsberg (7), there was no surgical procedure which guaranteed potential long-term success in returning the hindlimb to normal function (7). In addition, Schultz et al. (8) reported that, despite many clinical research reports comparing the different surgical techniques, only a few studies reached the standards for level I evidence (8). Level I studies provide data for valid decision-making and the ethical application of diagnostics and treatment. Moreover, in small animal orthopedics, level I evidence is reported to be optimally achieved by means of three evaluations or outcome assessment methods: force plate analysis, the surgeon's subjective and objective evaluation, and the pet owner's subjective evaluation (8). Although force plate analysis data offers an objective measurement, it evaluates the animal only at a specific moment in time and only regarding specific weight bearing on an affected limb, therefore constituting only one part of a larger clinical picture of intervention responses (9). Based on the results of the Hielm-Björkman studies (10, 11), a multifactorial questionnaire which focused on behavior and locomotion, completed by both a veterinarian and an owner would be better for evaluating chronic pain when compared with only a clinical evaluation carried out by an orthopedic veterinarian (10, 11).

Questionnaires for pet owners are already available in the veterinary practice for different pathologies, such as heart disease, spinal cord injuries, chronic pain, cancer kidney disease, dermatological disease and inflammatory bowel disease (11–23). Further clinical studies regarding veterinary orthopedics, such as the “COMP” (Canine Outcome Measures Program) and the “COI” (Canine Orthopedic Index), the “CBPI” (Canine Brief Pain Inventory), Helsinki Chronic Pain Index (HCPI) and other tools were developed and validated (21, 24–27). These client-reported tools are capable of assessing stiffness, gait, function and quality of life in dogs with osteoarthritis (27–31). All these reports considered only the owner assessment, and some of them were based only on phone interviews or email. These instruments have been validated to be indicative of the health level of dogs and useful for clinicians in obtaining a complete diagnosis and the correct therapy. In the same manner, patient-reported outcome devices are widely used in numerous fields of human medicine as they are considered to be valid and reliable (22).

To the best of the authors' knowledge, a combination of instruments to measure the phase of healing after canine CCL treatment does not exist. The development and validation of outcome instruments has been well-documented in human orthopedics. Different batteries of tests have been developed to assess rehabilitation status after cruciate ligament reconstruction in human medicine; however, only one exists regarding veterinary medicine (8, 31–36). The aim of these batteries of tests was to assess the functional outcome of knee/stifle rehabilitation and to optimize decision–making for a return to sports/activities by summarizing various tests.

In the present study, the authors evaluated a tool for the clinicians to easily record and assess the healing process in dogs surgically treated for CCL rupture. The tool, or device, is based on the collection of data at the time of the diagnosis of the CCL rupture and after surgical treatment, combining clinician and pet owner assessments, as has previously been suggested by several authors and by applying Evidence-Based Medicine (8, 10, 37).

This tool, the Bologna Healing Stifle Injury Index (BHSII), was developed as an evaluation sheet specifically edited for the assessment of stifle joint surgery. It was partially based on human medicine experience, where a questionnaire to assess patient opinion regarding their knees and associated problems is considered to have patient-relevancy, user-friendliness, reliability, validity and responsiveness to clinical change (38, 39).

The purpose of the present study was to develop and assess reliability, validity, and responsiveness of a novel tool (the BHSII), for the clinical evaluation of dogs with CCL rupture and healing after treatment. Combining clinician and owner assessments, the authors sought to validate a complete device useful for guiding clinical practitioners in their clinical decision-making.

## Materials and Methods

### Development of the BHSII

The BHSII (Supplementary Table 1) was composed of two parts: a survey for dog owners (BHSII-OQ: Owner Questionnaire) and a series of clinical evaluations concerning the orthopedic examination which the veterinarian utilized for the patient (BHSII-CR: Clinical Record).

The BHSII was developed in various steps between 2006 and 2010, following Brown's suggestions (9), and the data were collected at the Department of Veterinary Medical Sciences, University of Bologna, IT, from 2011 to 2018.

#### Step 1 – Setting the Questions

The owner multiple-answer survey was realized following and modifying the KOOS (Knee Injury and Osteoarthritis Outcome Score) questionnaire model used in human medicine to evaluate injury to the ligaments or menisci of the knee (38, 39).

The questions were modified to resolve the issue of the impossibility of direct application to canine patients as occurs in human medicine. The questions, or items, were designed to be as unambiguous and as clear as possible.

Once the set of items was formulated, the response method was chosen. The response method was selected as a Likert scale with five-levels of answers (0-never; 1-rarely; 2-sometimes; 3-often; 4-always). In order to allow a “no opinion” option, an odd number of answers was offered with scores ranging from 0 to 4, as suggested by Brown (9).

#### Step 2 – Selecting the Questions

A preliminary study was conducted involving owners and colleagues to test the questions several times. It was essential to assess whether each item was ambiguous or clear and easy to understand. Feedback of the owners were collected. The questions that needed explanation from a clinician, were poorly worded or irrelevant regarding the well-being of the dog, were reworded or deleted. At the end of this process a final list of items was selected.

The questions were grouped into domains.

The BHSII-OQ was divided into 3 domains relative to the behavior of dogs which revealed pain (P) (12 questions: P1 to P12), stiffness (S) (5 questions: S1 to S5), and function (F) (7 questions: F1 to F7), for a total of 24 items. Their aim was to evaluate the degree of pain, stiffness and function attributed to the dog's affected limb by means of the owner evaluation in reference to the previous 2 weeks as the period of time.

The BHSII-CR was directed to the veterinarians for their evaluations. It was made up of 2 domains: visual examination (V) (3 questions: V1 to V3) and manual examination of the joint treated (M) (7 questions: M1 to M7), for a total number of 10 items.

The visual examination domain referred to the presence or absence of lameness, abnormal gait, and their severity and frequency. The manual examination domain included questions regarding pain, patello-femoral crepitus, joint stability, range of motion, tumefaction, and muscle mass. The items of the Clinical Record were assessed as indicated in Supplementary Table 2. Radiographs of the injured stifle joint were taken for each dog at the time of first clinical evaluation, immediately after surgery, and at 1, 3, and 6 months postoperatively, but the osteoarthritis score was not collected and analyzed in the present study.

#### Step 3 – Data Collection

All answer scores were entered in an Excel spreadsheet. To allow a correlation among the domains, the sum of the total scores of each domain was normalized, transforming the values scored 0 to 4, and obtaining a value scale extending from 0 to 100 in which 0 indicated the presence of serious stifle joint problems and 100 the absence of any problems, according to the KOOS survey (39) (Tables 1,2).

**Table 1:** List of the domains, items, and the possible maximum score for each domain on a scale from 0 to 4.

**Domain**	**Sum of values of the following items**	**Maximum score for the domain**
Pain	P1–P12	48
Stiffness	S1–S5	20
Function	F1–F7	28
Visual examination	V1–V3	12
Manual examination	M1–M7	28

**Table 2:** BHSII Manual Score calculation: formula for obtaining a normalized score for each domain and the complete BHSII (scale from 0 to 100).

**Domain**	**Formula**	**Results**
Pain (P)	100–$\frac{\left(P1+P2+P3+P4+P5+P6+P7+P8+P9+P10+P11+P12\right)\times 100}{48}$	= BHSII Pain
Stiffness (S)	100 – $\frac{\left(S1+S2+S3+S4+S5\right)\times 100}{20}$	= BHSII Stiffness
Function (F)	100 – $\frac{\left(F1+F2+F3+F4+F5+F6+F7\right)\times 100}{28}$	= BHSII Function
Visual examination (V)	100 –$\frac{\left(V1+V2+V3\right)\times 100}{12}$	= BHSII Visual Ex.
Manual examination (M)	100 – $\frac{\left(M1+M2+M3+M4+M5+M6+M7\right)\times 100}{28}$	= BHSII Manual Ex.
BHSII	100 – $\frac{total\;score\;of\;the\;complete\;questionnaire\times 100}{136}$	= BHSII Total Score

*The formula required is: normalized score = 100 –* $\frac{total\;score\;of\;each\;subscale\times \mathrm{100}}{maximum\;possible\;score\;for\;the\;subscale.}$

* maximum possible score for the domain as reported in Table 1.

#### Step 4 – Clinical Study

A clinical study was designed to assess the reliability, validity, and applicability of the BHSII. A prospective study was conducted on dogs having a diagnosis of CCL rupture with a plan for surgical treatment carried out by the same surgeon in order to limit the variations based on the different skills of many surgeons (Surgical Group-SG). A control group of healthy dogs (CG) was enrolled instead of a placebo group for ethical reasons.

Dogs with a history of lameness, undergoing an orthopedic examination with a diagnosis of unilateral CCL rupture, without concomitant orthopedic problems were included. The owners had to give written informed consent and they agreed to the participation of their dog to the study and to bring their dog to be rechecked after surgery. The medical records included the written informed consent form signed by every dog owner as required for treatment of the data collected. Furthermore, the local ethical committee did not require the approval for this research protocol: the application of the surveys does not produce any pain in the animals included in the study, and they were otherwise normally threated for their pathology, with the same clinical routine as if they were not included in the study.

No restrictions of enrollment regarding breed, gender, age, and weight were applied.

The BHSII for the SG was completed at the time of diagnosis (T0) and at 1, 3, and 6 months after surgery (T1, T3, and T6, respectively), by the dog owners (BHSII-OQ) and by the veterinary surgeon who reached the diagnosis (BHSII-CR). While for the CG the BHSII was administered twice in 2 weeks for statistical purposes.

Furthermore, the dogs of the SG were divided into four groups based on age (Age Group-AG) to better assess the feasibility of the test: Group A (<3 years), Group B (4–6 years), Group C (7–9 years) and Group D (>9 years).

### Statistical Analyses and Validation Tests

Twenty healthy dogs, with no history of right stifle disease, and free from any other orthopedic condition in any limb at the moment of the surveys, were enrolled to complete the BHSII twice, using them as the CG.

The demographic data of the dogs of both the CG and the SG were reported as mean and standard deviations (SD). The weight of the dogs with CCL rupture was evaluated for normal distribution using a Shapiro-Wilk test and compared between groups using a Kruskall-Wallis test. The normalized value of the BHSII total scores, of the BHSII-OQ, of the BHSII-CR and of each domain of the dogs were summed up and reported as median and interquartile range (IQR) at every postoperative exam (from T0 to T6 months). Categorical variables (gender and limb involved) were compared using a Chi squared test.

All analyses were carried out with the use of statistical software (MedCalc® Software 16.8.4, Ostend, Belgium; GraphPad Software Prism7, Inc., San Diego, CA) and values of *P* < 0.05 were considered significant.

Based on the suggestions given by Brown (9), the questionnaire underwent reliability and validity tests as reported below. At the moment of the tool construction and application to this cohort of dogs, no other validated instruments for the measurement of the same attribute was existing, so internal multiple analyses and assessments where conducted (9).

#### Test-Retest Reliability

The stability of the tool was determined by test-retest reliability comparing the results of each domain of the questionnaire collected 2 weeks apart from the CG using an intraclass correlation coefficient (ICC) test. Two weeks were considered to be a reasonable period: short enough to avoid any significant changes in the normal dogs health status, but at the same time long enough to permit to the owner and the clinician not to remember the first questionnaire scores. This was consistent with other studies available in literature, as Ross et al. (38) which repeated the test after 9 days, Lavan (19) who used a period of 2 weeks and in Brown (9) it is suggested to repeat the questionnaire after 7 days (9, 19, 38). An ICC ≥ 0.7 was considered suggestive of a high probability of obtaining a similar retest in the same animal (40).

In the SG, the internal consistency of the BHSII-OQ was evaluated item by item at T0 using the Cronbach-α test. A Cronbach-α score >0.7 was considered indicative of the good internal consistency of the BHSII-OQ (41).

#### Construct Validity

A receiver operating characteristic (ROC) curve was used to determine the specificity and sensitivity of the questionnaire to differentiate healthy dogs (CG) from those with CCL rupture (SG at T0). An area under the curve (AUC) >0.7 was considered suggestive of moderate accuracy of the questionnaire (42).

An objective correlation was tested between the outcome measurements of the owner evaluation and the clinician assessment. The scores of each domain, BHSII-OQ, BHSII-CR, and of the total BHSII were correlated with each other using the Spearman's correlation coefficient. The correlations were classified based on British Medical Journal recommendations: *r*_*s*_: 0–0.19 very weak, 0.2–0.39 weak, 0.40–0.59 moderate, 0.6–0.79 strong, and 0.8–1 very strong (43).

#### Responsiveness

A Wilcoxon paired test was used to compare the total BHSII, the BHSII-CR, and the BHSII-OQ scores, and the scores of each domain obtained from the SG over time. A Kruskal-Wallis test was used to compare the same scores between the AGs at each time point.

## Results

Twenty healthy dogs of different breeds were considered as the CG; there were 4 males and 16 females. Their mean age was 5.8 ± 3.3 years and their mean body weight was 23.4 ± 11.3 kg. All limbs evaluated were from the right side.

A total of 158 dogs of different breeds with CCL rupture were included in the study as the SG. The most represented in the SG were cross-breed dogs (*n* = 52), followed by the Labrador Retrievers (*n* = 17), Boxers (*n* = 10), American Staffordshire Terriers (*n* = 8), German Shepherds (*n* = 8), Rottweilers (*n* = 8), and Cane Corso dogs (*n* = 7). Other breeds were represented in the study cohort with a number of cases inferior to 5. There were 65 males and 93 females the mean age was 6.3 ± 3 years and the mean body weight was 29.2 ± 14.4 kg; the stifle joints involved were 78 right and 80 left stifles.

In the AGs, the mean body weight was 31.1 ± 13.0 kg in Group A (*n* = 39), 34.7 ± 13.3 kg in Group B (*n* = 40), 28.8 ± 16.2 kg in Group C (*n* = 40) and 22.2 ± 12.2 kg in Group D (*n* = 39). The weight of the dogs in Group B was statistically significantly higher as compared with those in Groups C and D while the weight of the dogs in Group D was statistically significantly lower as compared with that of Group A. There was no statistically significant difference in gender distribution among the groups.

A total of 40 BHSII sheets of the CG and 555 sheets of the SG were tested and analyzed. All 158 dogs included in the SG were evaluated up to 3 months after surgery (T3) while 77 participants did not complete the BHSII-OQ and did not bring their dogs for clinical evaluation at T6.

### Test-Retest Reliability

In the CG, the BHSII-OQ, the BHSII-CR and the BHSII had an ICC ≥ 0.9: the likelihood of obtaining a similar response in the same animal within 2 weeks and without any changes occurring in the animal's status was considered high.

Therefore, the domains revealing pain (ICC = 0.96) stiffness (ICC = 0.96) and function (ICC = 0.92), and those referring to the visual examination (ICC = 1) and manual examination (ICC = 0.94) were all considered in the subsequent clinical survey.

The Cronbach-α for the 24 items included in the BHSII-OQ was 0.84, and was considered indicative of good internal consistency.

### Construct Validity

A ROC curve analysis was used to test the ability of the BSHII to differentiate between healthy dogs and those with CCL rupture. On the basis of the ROC curve the questionnaire had high accuracy (AUC = 0.92). A questionnaire score >27 and ≤ 85.6 should predict the health level of dogs with CCL rupture with 100% sensitivity [95% confidence interval [CI]: 97.7–100%] and 100% specificity (95% CI: 83.2–100%).

Correlation between the BHSII-OQ and the BHSII-CR at T0 was found (convergent construct validity) which indicated congruence between the owners' opinion and the clinical assessment (*r*_s_ = 0.3635; *P* < 0.0001). All the correlations domain-to-domain and domain-total score (BSHII) were found to be convergent except for pain during the manual examination (Table 3).

**Table 3:** Domain-to-domain and domain-total correlation (Spearman's correlation coefficient).

**Domain**	**Pain**	**Stiffness**	**Function**	**Visual**	**Manual**	**BHSII-OQ**	**BHSII-CR**
Pain	NA	0.4582^‡^	0.4967^‡^	0.2894^†^	0.1219^*^||	0.8583^§^	0.3635^†^
Stiffness	0.4582^‡^	NA	0.5181^‡^	0.1706^†^	0.2050^‡^	0.7276^§^	0.2390^†^
Function	0.4967^‡^	0.5181^‡^	NA	0.5013^‡^	0.1909^†^	0.8116^§^	0.2504^†^
Visual	0.2894^†^	0.1706^†^	0.5013^‡^	NA	0.1608^†^	0.3980^‡^	0.5568^‡^
Manual	0.1219^*^||	0.2050^†^	0.1909^†^	0.1608^*^	NA	0.2139^†^	0.8882^§^
**TOOL**							
BHSII-OQ	0.8583^§^	0.7276^§^	0.8116^§^	0.3980^‡^	0.2139^†^	NA	0.3635^†^
BHSII-CR	0.3635^†^	0.2390^†^	0.2504^†^	0.5568^‡^	0.8882^§^	0.3635^†^	NA

* Very weak correlation (0–0.19).

† Weak correlation (0.2–0.39).

‡ Moderate correlation (0.4–0.59).

§ *Strong and very strong correlation (0.6–1) significant per p < 0.05. ||Not significant. NA, Not Applicable*.

### Responsiveness

In the SG, the BSHII, the BSHII-OQ, and the BSHII-CR scores, and the scores of each domain increased significantly postoperatively at each time point as compared with baseline. In addition, by the first postoperative month, the scores improved significantly over time at each time point when compared with each other (*P* < 0.05). The median and IQR are listed in Table 4.

**Table 4:** Scores of the tool and of each domain obtained from 158 dogs with CCL rupture before (preoperative) and after surgical treatment.

	**T0 (*n* = 158)**	**T1 (*n* = 158)**	**T3 (*n* = 158)**	**T6 (*n* = 81)**
**TOOL**				
BHSII-OQ	59.37 (47.91–71.88)	82.29 (72.92–90.62)	94.79 (87.50–96.88)	97.92 (94.80–99)
BHSII-CR	58.33 (52.5–65)	80 (70–86.11)	90 (82.50–95)	92.5 (88.89–97.50)
BHSII	59.56 (50.76–67.65)	81.62 (74.24–88.24)	92.65 (86.36–95.59)	96.32 (93.23–98.53)
**DOMAIN**				
Pain	70.83 (58.33–81.25)	87.5 (77.1–93.75)	95.83 (91.67–97.92)	97.92 (95.83–100)
Stiffness	55 (40–75)	80 (70–90)	90 (80–100)	95 (90–100)
Function	42.86 (28.57–60.71)	78.57 (67.86–89.3)	94.65 (85.71–100)	100 (96.43–100)
Visual	50 (41.67–58.33)	66.67 (66.67–83.33)	100 (83.33–100)	100 (100–100)
Manual	64.29 (54.17–71.43)	82.14 (71.43–89.29)	87.5 (79.17–92.86)	91.67 (83.33–96.43)

*Data are reported as median and interquartile range (IQR). For all tools and all domains the scores obtained at each time point post-operatively differed significantly from the preoperative scores (P < 0.05). T0: preoperative time. T1, T3, and T6: 1, 3, and 6 months postoperative, respectively*.

Responsiveness was evaluated among the AGs; at baseline, the BHSII score was significantly higher for the dogs in Group A as compared with the other three groups. At the same time, score of the pain domain in the in the BSHII-OQ was significantly higher in Group A as compared with the other three groups. At T1, T3, and T6, the questionnaire scores did not differ significantly between groups.

## Discussion

In this paper, the authors developed a questionnaire designed to assess the healing of dogs following the surgical intervention for CCL rupture. Other surveys were already available in the orthopedic veterinary field, such as the CBPI, the HCPI and the LOAD (11, 20, 24, 25, 27), however none of these is focused on the healing and health status of dogs which underwent a surgical treatment for cranial cruciate ligament (CCL) rupture. The most similar to the questionnaire presented in this study is probably the CBPI (24, 27) which evaluates the pain in dogs affected by osteoarthritis. However, the main difference between the two is that the BSHII was developed combining owner and clinician assessments, while the CBPI (as all the other questionnaires present in literature) is only focused on the owner answers. Furthermore, the response method selected for this tool was a five-level scale (from 0 to 4) in order to allow a “no opinion” option as suggested by Brown (9), while in the other survey an even number of options was provided (9). Another difference between the two is that the questions directed to the owners in the present tool are more numerous, being more precise and restrictive.

In the first part of the study, the internal consistency, stability, and validity of the questionnaire were evaluated using a stepwise approach. In the CG the ICC suggested high reliability of each domain and, therefore, they were all considered valid when evaluating the healing process of surgically treated dogs and were all taken into consideration in developing the tool.

The Cronbach-α suggested a high correlation of the items within the BSHII-OQ and, therefore, a high degree of agreement in expressing the same concept within the owner questionnaire. On the contrary, the BHSII-CR was made up of two domains which were recognized as pivotal steps of the orthopedic examination and, therefore, the evaluation of its internal consistency was not carried out. Moreover, on the basis of the ROC analysis, the questionnaire was considered to have high accuracy in differentiating healthy dogs from those with CCL rupture.

The convergent validity between the owner evaluation and the clinical examination was tested and were found to be correlated (*r*_*s*_ = 0.3635; *P* < 0.0001).

As expected the correlation between BHSII-OQ and BHSII-CR was weak. The BHSII-OQ is the opinion of the owners which reflects the health status of their dogs, despite this, the owner evaluation at each time point had the same trend as the clinical examination, thus providing significant information to the clinician. This finding was in accordance with a previous study in which the owner assessment was found to be a reliable and responsive method for assessing outcome in CCL rupture (44). Moreover, as Swinscow and Campbell (43) stated, the classification of the correlations had rather arbitrary limits, and the context of the results should be considered (43).

All these steps were based on the suggestions given by Brown (9). In that study it is explicitly indicated that whereas other validated tools developed to measure the same attribute are available, the easiest and most direct way to validate the instrument is to administer it with the already existing one and test the results correlation between them. On the other hand, when this is not possible, construct validity must be tested differently. To the best of the authors' knowledge, at the moment of the tool construction and application to this cohort of dogs, there were no other validated instruments for the measurement of the healing after cranial cruciate ligament surgery; the most similar questionnaires validated in the years were the CBPI, the HCPI and the LOAD (11, 20, 24, 25, 27), but not all of them were available before the administration of our questionnaire to our dogs, and anyway their target was different than ours. For these reasons it was not possible to compare this tool to others and internal multiple assessments where conducted. Furthermore, Brown (29–31) tested the COI in three steps and did not compare it with other available questionnaires. For example, to test its responsiveness, the same tool was administered to different groups of owners of dogs treated differently for osteoarthritis (non-steroidal anti-inflammatory drugs or placebo). The same kind of study was conducted in 2008 by Brown et al. for the CBPI (24). In our case, this was not possible, as the questionnaire is focused on the healing after surgical treatment, so the comparison with a placebo treatment could not be considered.

In the present study, dogs of the SG were divided into various age groups. In fact, changes in health status and behavior are commonly observed in dogs due to natural and progressive aging (19). Therefore, the grouping was carried out to verify whether domains and items were statistically significantly different between dogs of different ages. The total score of the tool at baseline was higher in younger dogs (Group A) as compared with the other groups, the difference might have been due to the score of the domain relative to pain evaluation which, at the same time, was higher in Group A. When assessing pain in animals, age and previous experience must be taken into consideration since they can influence the behavior toward painful stimuli or toward daily activity (45, 46). Furthermore, younger dogs are less likely to experience a maladaptive pain state due to chronic osteoarthritis and they might have a higher tolerance to pain (47).

The tool was applied in the SG as a postoperative follow-up. One month postoperatively, the scores increased significantly across time as compared with the baseline. Those improvements are the result of the owner and the clinician evaluations summed up representing a statistically significant difference but also a clinical improvement (48, 49). In the present study, for example, the mean normalized score of the manual examination domain increased from 64.29 to 82.14, from T0 to T1, respectively, until 91.67 at T6 that means absence of any problem in the scale from 0 to 100 (Table 4).

All the domains of the BSHII-OQ had very weak or weak correlations with the domains of the BSHII-CR with the exception of the correlation between function and visual which was moderate.

As expected, the domains of the BHSII had good convergent correlations among each other, except for the pain assessed by the pet owner and the manual examination for which the correlation was not significant. It is possible that some owners were not able to assess their pet's pain behaviors correctly, however it is well-known, for example, that many dogs with osteoarthritis of a joint show pain behaviors but will not react upon palpation (26, 50, 51). Chronic pain can be evaluated by means of some behavioral changes, such as lethargy, decreasing activity, demeanor and social behavior, which are apparent only to someone very familiar with the dog (52). It is also known that pain can condition indirectly other items such as range of motion, lameness, and muscle mass, or it can be conditioned by tumefaction, effusion, crepitus and joint instability. On the other hand, the pain is only a part of a larger clinical picture that may explain a not significant correlation between manual examination (domain of BHSII-CR) and pain (domain of BHSII-OQ) (45, 53). In addition, the results in the present study were in accordance with a study in which the authors did not find any correlation between the owner and the clinician pain assessments (10). The authors concluded that the owner and the veterinarian should collaborate in optimizing pain assessment (10). Pain is difficult to determine in dogs; thus, in a study regarding a relationship between pain and lameness, the results obtained from the owner questionnaire and those obtained from force plate analyses were combined. The authors concluded that the owner questionnaire could help the clinician to determine the degree of lameness when a force plate was not available (50).

The domains related to manual examination had a positive but weak correlation with function and stiffness. These domains revealed signs obtained by means of two different approaches to evaluation: palpation as the first and observation as the second. Despite this statistical result, in clinical practice, some of the items of the manual examination domain, i.e., range of motion, effusion, and crepitus, may be causes of stiffness, and may therefore influence the functionality of the limb causing little to no associated pain (51, 53, 54).

The very strong correlations of the pain, stiffness and function domains with the total score showed the influence of the owner evaluation on the BHSII, greater than that of the clinical examination which supported the aim of creating an instrument which showed the large picture of the healing process after treatment for CCL rupture.

It is interesting to note that the IQR of all means considered appear to narrow over time. This could mean that at T0 there is wide score variability (i.e., chronic vs. acute symptoms, several degrees of lameness, pain, swelling, …), while the reduced IQR at T6 could depend on the normalization of health status and the completion of the healing process. Breeds were not statistically matched between SG and CG as the 20 healthy dogs were used only for test-retest reliability purposes, so they were not directly confronted to the study cohort. Furthermore, the questionnaire has been developed to be valid for all the canine patients.

A limitation of the study was that the BHSII did not take into consideration the radiographic examination of the affected joint. The radiographic exam is an important part of the orthopedic evaluation; however, it has been reported that the osteoarthritis radiographic findings do not always correctly depict the functional and clinical aspect of the limb (55). Furthermore, it would be interesting to carry out an additional study including only dogs without osteoarthritis at T0 in order to evaluate the validity of the considerations above and the importance of the osteoarthritis evolution in the total score of the tool.

In conclusion, the analysis conducted on the BHSII confirmed its good reliability, validity and responsiveness when applied to evaluating the healing of dogs treated for CCL rupture. The BHSII is statistically valid in measuring the level of healing, providing an instrument which, by means of the combination of the owner and the clinician evaluations, may improve the ability of the practitioner to assess the progress of the healing process of the canine patient during the postoperative period.

The results of this study should be considered of primary importance in assessing the response to surgical treatment of stifle injuries with respect to quality of life. The use of the BHSII could be contemplated for future studies regarding new surgical procedures or about the ones already commonly used; this tool could facilitate the data collection and their comparisons, improving at the same time their level of evidence.

## Data Availability

The datasets generated for this study are available on request to the corresponding author.

## Ethics Statement

The study was conducted in a manner consistent with the Animal Welfare Acts and did not require the ethics committee approval.

## Author Contributions

SP: conceptualization, study design, and manuscript writing. CL: statistical analyses and manuscript editing. LG: data collection and manuscript editing. NR: study design and manuscript revision.

### Conflict of Interest Statement

The authors declare that the research was conducted in the absence of any commercial or financial relationships that could be construed as a potential conflict of interest.

## Abbreviations

AG: Age Group
AUC: area under the curve
BHSII: Bologna Healing Stifle Injury Index
BHSII-OQ: Bologna Healing Stifle Injury Index - Owner Questionnaire
BHSII-CR: Bologna Healing Stifle Injury Index-Clinical Record
CG: Control Group
KOOS: Knee Injury and Osteoarthritis Outcome Score
ICC: Intraclass Correlation Coefficient
IQR: Interquartile Range
ROC: Receiver Operating Characteristic
SG: Surgical Group.
