# Supplementary material for: Evidence-Based Veterinary Medicine: A Tool for Evaluating the Healing Process After Surgical Treatment for Cranial Cruciate Ligament Rupture in Dogs
Source: Front Vet Sci. 2019 Mar 5;6:65. doi: 10.3389/fvets.2019.00065 (PMC6411761; doi:10.3389/fvets.2019.00065)
Supplement: Supplementary file 1 [file Table_1.pdf]

Supplementary Material

Evidence-Based Veterinary Medicine: a tool for evaluating the healing process after surgical treatment for cranial cruciate ligament rupture in dogs

Stefania Pinna*, Carlotta Lambertini, Lisa Grassato, Noemi Romagnoli

*** Correspondence:** Dr. Stefania Pinna: stefania.pinna@unibo.it

Supplementary Table 1– The BHSII (Bologna Healing Stifle Injury Index) is composed of two parts: the BHSII-OQ (BHSII – Owner Questionnaire) which consists of 3 domains with a total of 24 items and the BHSII-CR (BHSII – Clinical Record) which consists of 2 domains with a total of 10 items. Each item can be answered by a 5-point scale (0 to 4).

The forms have to be compiled by the pet-owner (part 1) and by the veterinarian (part 2), with a score from 0 (no problem) to 4 (severe alteration).

**BHSII (Bologna Healing Stifle Injury Index)**			
Date (DD-MM-YY)		Study ID	
Owner (Surname – Name)		Phone	
Breed	Gender M F	Name of Dog	
Diagnosis	Limb R L	Age	Weight kg
Surgical technique		Operative time days	

Part 1. BHSII-OQ (Bologna Healing Stifle Injury Index – Owner Questionnaire)

Directions for the owner: for each of the following questions, circle the number which best reflects the condition of your dog in the past two weeks.

		SCORE				
**PAIN**		Never (0)	Rarely (1)	Sometimes (2)	Often (3)	Always (4)
P1.	Has your dog changed its habits (i.e. where it sleeps, invitations to play, requests for walks) (answer affirmatively even for 1 change among those listed)?	0	1	2	3	4
P2.	Has it changed its response to everyday orders?	0	1	2	3	4
P3.	Does it need to sit down after walks usual distance?	0	1	2	3	4
P4.	Does it need to sit down after walks for bodily functions?	0	1	2	3	4
P5.	Does it change its behavior when the weather changes?	0	1	2	3	4
P6.	Does it lick or bite its stifle joint?	0	1	2	3	4
P7.	Has there been swelling in the stifle joint over the past two weeks?	0	1	2	3	4
P8.	Does it have difficulty going up stairs?	0	1	2	3	4
P9.	Does it have difficulty going down stairs?	0	1	2	3	4
P10.	Does it have difficulty getting in the car?	0	1	2	3	4
P11.	Does it have difficulty sitting down (i.e., turning around repeatedly)?	0	1	2	3	4
P12.	Does it whimper when it makes certain movements (lying down, getting up, turning around)?	0	1	2	3	4
**STIFFNESS**		Never (0)	Rarely (1)	Sometimes (2)	Often (3)	Always (4)
S1.	Does your dog exhibit stiffness of movement in the morning or after a period of rest?	0	1	2	3	4
S2.	Does it exhibit stiffness of movement after a long walk?	0	1	2	3	4
S3.	When it is sitting down, does it hold the affected limb in an abnormal position?	0	1	2	3	4
S4.	Does it move the affected limb in an abnormal way?	0	1	2	3	4
S5.	What is its position when urinating: if male, does it put its weight on the affected limb? If female, does it crouch down normally?	0-Always	1-Often	2-Sometimes	3-Rarely	4-Never
**FUNCTION**		Never (0)	Rarely (1)	Sometimes (2)	Often (3)	Always (4)
F1.	Does your dog limp when walking on soft ground (i.e. lawn, sand)?	0	1	2	3	4
F2.	Does it limp when walking on hard ground (i.e. asphalt, cement)?	0	1	2	3	4
F3.	Does it make abnormal movements when sitting down?	0	1	2	3	4
F4.	Does it make abnormal movements when getting up?	0	1	2	3	4
F5.	Does it behave strangely when circling around?	0	1	2	3	4
F6.	How frequent is the limp?	0 – no limping at any time<br>1 – normal most of the time. Slight limp after rest/prolonged exercise<br>2 – normal 50% of the time. Moderate limp after rest/prolonged exercise<br>3 – normal for brief periods. Limping most of the time<br>4 – limping all the time				
F7.	What type of limp is it?	0 – no limping at any time<br>1 – slightly abnormal gait, puts steady weight on the limb<br>2 – clearly abnormal gait, puts steady weight on the limb<br>3 – seriously abnormal, occasionally does not put weight on the limb when in movement<br>4 – never puts weight on the limb at any time				

**Part 2. BHSII-CR (Bologna Healing Stifle Injury Index – Clinical Record)**					
Directions for the clinician: for each of the following items circle the number which reflects the severity of the signs revealed during the orthopedic examination.					
	**SCORE**				
**VISUAL EXAMINATION**					
V1. Lameness	0 – none<br>1 – slight: slightly altered movement, function preserved<br>2 – altered movement, function preserved<br>3 – moderate: altered movement, function impaired<br>4 – severe: altered movement, function lost				
V2. Gait in which limping is seen	0 – no limping at any gait<br>1 – limping only when running<br>2 – limping when trotting<br>3 – limping even when walking (i.e., in all gaits)<br>4 – does not put weight on limb at any gait				
V3. Abnormal gait (i.e. asymmetrical movement and/or jerky gait)	0 – none	1 – slight	2 – mild	3 – moderate	4 – severe
**MANUAL EXAMINATION**					
M1. Pain: palpation and passive movement	0 – none	1 – slight	2 – mild	3 – moderate	4 – severe
M2. Patello-femoral crepitus	0 – none	1 – slight	2 – mild	3 – moderate	4 – severe
M3. Cranial drawer test	0=stable	1=1-3mm	2=4-6mm	3=7-9mm	4=10-12mm
M4. Range of motion in extension	0=162°-158°	1=157°-153°	2=152°-148°	3=147°-143°	4=142°-138
M5. Range of motion in flexion	0=41°-45°	1=46°-50°	2=51°-55°	3=56°-60°	4=61°-65°
M6. Tumefaction-effusion-edema	0 – none	1 – slight	2 – mild	3 – moderate	4 – severe
M7. Muscle mass in thigh region	0 – normal	1 – almost normal	2 – reduced	3 – seriously reduced	4 – atrophic

These sheets can be used freely in clinical practice and research; for any other use (e.g., commercial use), please contact the authors.
