# Supplementary material for: Evidence-Based Veterinary Medicine: A Tool for Evaluating the Healing Process After Surgical Treatment for Cranial Cruciate Ligament Rupture in Dogs
Source: Front Vet Sci. 2019 Mar 5;6:65. doi: 10.3389/fvets.2019.00065 (PMC6411761; doi:10.3389/fvets.2019.00065)
Supplement: Supplementary file 2 [file Table_2.pdf]

Supplementary Material

Evidence-Based Veterinary Medicine: a tool for evaluating the healing process after surgical treatment for cranial cruciate ligament rupture in dogs

Stefania Pinna*, Carlotta Lambertini, Lisa Grassato, Noemi Romagnoli

*** Correspondence:** Dr. Stefania Pinna: stefania.pinna@unibo.it

Supplementary Table 2 – The explanation of how the items of the BHSII-CR are assessed by the veterinarian following the steps of orthopedic examination.

Guidelines for orthopedic examination		
**ITEM**		
	**VISUAL EXAMINATION**	
V1. V2. V3	Lameness/abnormal gait	Assessed by means of the observation of the gait of the animal when walking, trotting and running, graded on a scale from 0 to 4
	**MANUAL EXAMINATION**	
M1.	Pain	Assessed by means of the patient's reaction to palpation and passive movement of the stifle joint
M2.	Patello-femoral crepitus	Assessed by means of palpation and passive movement
M3.	Articular stability	Assessed by means of the drawer test, measured from 0 to 12 mm
M4.M5.	Range of motion	In extension and flexion, compared to the contralateral limb; measured in unsedated patients with a plastic sexagesimal goniometer, using 41°-162° as the reference range
M6.	Periarticular tumefaction	Synovial effusion and edema assessed by means of palpation
M7.	Muscle mass of the thigh	Quantified by means of palpation and measurement of the circumference as compared to the contralateral limb
